# Predictive value of [-2]propsa (p2psa) and its derivatives for the prostate cancer detection in the 2.0 to 10.0ng/mL PSA range

**DOI:** 10.1590/S1677-5538.IBJU.2016.0256

**Published:** 2017

**Authors:** I. Vukovic, D. Djordjevic, N. Bojanic, U. Babic, I. Soldatovic

**Affiliations:** 1 Clinic of Urology, Clinical Center of Serbia, School of Medicine, University of Belgrade, Serbia;; 2 Institute of Medical Statistics and Informatics, Belgrade, Serbia

**Keywords:** Biomarkers, Prostatic Neoplasms, Prostate-Specific Antigen

## Abstract

**Introduction:**

To assess predictive value of new tumor markers, precursor of prostate specific antigen (p2PSA) and its derivates-%p2PSA and prostate health index (PHI) in detection of patients with indolent and aggressive prostate cancer (PC) in a subcohort of man whose total PSA ranged from 2 to 10ng/mL.

**Materials and Methods:**

This cross-sectional study included 129 consecutive male patients aged over 50 years, with no previous history of PC and with normal digital rectal examination findings, but with serum PSA in interval between 2 and 10ng/mL. All patients underwent standard transrectal ultrasonography guided prostate biopsy for the first time. For all patients, serum PSA, free PSA (fPSA) and p2PSA were measured and PHI and %p2PSA were calculated.

**Results:**

PHI and %p2PSA levels were significanlty higher in patients with PC compared to those without this malignancy. The same findings have been observed in group of patients with Gleason score ≥7 compared to those with Gleason score <7. ROC analysis reveled the highest area under the curve with these two markers. Multivariate logistic regression showed significant improvement in PC detection and its agressive form (assumed as Gleason score ≥7).

**Conclusions:**

New markers, derivates of p2PSA (especially %p2PSA and PHI), represente potentially very important clinical tool for predicting presence of PC, and even more important, to discriminate patients with Gleason score <7 from those with Gleason score ≥7 with total PSA in range from 2 to 10ng/mL.

## INTRODUCTION

Prostate cancer (PC) is the fifth leading cause of cancer in male population worldwide. In western countries, it represents the most commonly diagnosed cancer in men. Autopsy studies highlighted the fact that the prevalence of PC in men 70 years of age or older is around 80% (1-3).

Prostate-specific antigen (PSA) is widely known as a main serum biomarker for the early detection of PC (4). Namely, its introduction in routine urological clinical practice in the early 1980s deeply influenced PC diagnosis and management, with a consequent reduction in PC-related mortality during the past three decades (5-7).

However, keeping in mind the fact that PSA is an organ-specific but not cancer-specific marker, numerous limitations could appear during evaluation of this screening test validity. Firstly, it has been recognized that PSA has a low specificity, with the positive predictive value around 25%, leading to a huge number of false-positive results and up to 75% unnecessary prostate biopsies. Secondly, PSA also has low sensitivity, with about one-third of all PC cases with the level of this marker below the value of 4ng/mL. Finally, the findings from numerous studies have been highlighted that almost 60% of all PC operative treated patients had so-called indolent tumors, characterized with low malignant potential (8, 9). Keeping in mind this fact, it could be hypothesized that majority of these patients were over-detected and subsequently over-treated. All these facts clearly pointed out that PSA alone has no satisfied predictive value in PC detection.

Consequently, in more recent years, considerable efforts have been made to find new specific markers for early PC detection with improved potential to detect its aggressive clinical form. In this line, the introduction of several PSA derivatives (free PSA [fPSA], percentage of free PSA [%fPSA], PSA density, PSA velocity, Prostate health index [PHI],) in clinical practice significantly improved the accuracy and validity of PSA in identifying PC. Moreover, fPSA was found to include several subforms, such as a precursor form of PSA (proPSA). Theoretically, seven isoforms of proPSA should exist of which [-2] proPSA (p2PSA) is the most stable form. The results from several studies suggested that p2PSA has the highest specificity in PC detection (10, 11). It originates mainly from malignant prostate epithelium, especially in periphery zone of prostate, which is the dominant location of cancer occurrence (12, 13). Therefore, nowadays this marker represents the most promising tool for early PC detection. Additionally, it has been shown that p2PSA is also capable to make distinguish between clinically insignificant tumor (low grade) and cancer that needs to be treated.

Keeping in mind all mentioned above, the objective of this study was to *assess predictive value of* tumor markers p2PSA and its derivates, %p2PSA, and PHI in detection of patients with aggressive PC (assumed as Gleason score ≥7) in a sub-cohort of men whose total PSA ranged from 2 to 10ng/mL.

## MATERIALS AND METHODS

### Design, setting and participants

Study was conducted in Clinic of Urology, Clinical Center of Serbia, Belgrade, from January 2012 to January 2014. This cross-sectional investigation included 129 consecutive patients who underwent prostate biopsy for the first time. Inclusion criteria were: age over 50 years, no previous history of PC, normal digital rectal examination findings, serum PSA in interval between 2 and 10ng/mL, and minimally 12 biopsy cores taken from patient. Exclusion criteria were: previous consumption of medications that influence on PSA level (Finasteride, Dutasteride), previous surgical intervention on prostate (Transurethral prostatectomy TURP, biopsy), acute prostatitis, urinary tract infection, and previous androgen therapy.

Study was approved by Ethic Committee of Clinical Center of Serbia and Faculty of Medicine, University of Belgrade. All patients were completely informed about procedure and possible complications. Written consent was obtained from all patients.

### Interventions, measurement and data collection

At first examination, complete patient history (urological and general) and urological examination was done. Subsequently, blood samples were drawn and immediately stored in refrigerator at 4ºC temperature. Serum samples from whole blood were obtained by centrifuge and stored at-20ºC. When all samples were collected, serum PSA, fPSA and p2PSA were measured. Our laboratory routinely measures only serum PSA levels by Abbott test with CMIA technique. Access Hybritech assays (Backman Coulter Inc., Brea, CA, USA) were used to measure serum PSA, fPSA and p2PSA. p2PSA is measured using Hybertech p2PSA automated immunoassay. Hybritech calibrations were used for PSA and fPSA levels. After obtaining p2PSA, fPSA and PSA results, these were combined to calculate PHI:

PHI = (p2PSA/fPSA) x (square root of PSA) (equation 1)

In addition, %p2PSA was calculated using following formula:

%p2PS = p2PSA / (fPSAx1000) x 100. (equation 2)

Blood analysis also included C-reactive protein (CRP), serum protein and testosterone.

Physical examination comprised digital rectal examination. Furthermore, all participants underwent standard transrectal ultrasonography guided prostate biopsy. Minimal 12 cores biopsies were taken. Six cores were taken from peripheral zone of each lobe, 2 of those cores were form apex, 2 from middle part and 2 from base of prostate.

Preparation of biopsy core and microscopically examination was done in Department of Pathology, Clinical Center of Serbia. Biopsy specimens were placed in specific single-core specimen containers and then processed and evaluated by experienced genitourinary pathologist. Prostate cancer was identified and graded according to International Society of Urological Pathology definitions (14).

Pathological findings were divided into two groups, with and without PC. Findings of patients with confirmed cancer were further investigated to calculate Gleason score. Afterward, patients with cancer were divided into subgroups depending on Gleason score, patients with score less than 7 and patients with 7 and higher Gleason score.

### Statistical analysis

Data are presented as counts (percents), mean±sd or median (25th-75th percentile), depending on data type and distribution. T test and Mann-Whitney U test tests were used for group comparisons. Receiver operating characteristics (ROC) area under the curve (AUC) was used to assess significant marker of PC and to determine cut-off value. Univariate and multivariate logistic regression were used to fit prediction of PC by explanatory variables. Hosmer-Lemeshow test was used to check for goodness of fit of logistic regression model (calibration of the model). All statistical analyses were performed in SPSS 20.0 (IBM corp.) statistical software. All p values less than 0.05 were considered significant.

## RESULTS

Study included 129 patients, 65 with PC (50.4%) and 64 without PC (49.6%). Basic clinical characteristics of the study population are presented in Table-1. Significant differences between examined groups were observed in fPSA, %fPSA, %p2PSA and PHI. There were no significant differences between groups in respect of values of proteins, CRP and testosterone. Furthermore, mean age was also very similar in both groups.

**Table 1:** Basic characteristics, labaratory and Prostate Specific Antigen.

	PCa	Non-PCa	p value
Age	65.3±6.6	64.0±6.6	0.281
tPSA	5.81±1.98	6.24±1.96	0.220
fPSA	0.84±0.46	1.21±0.62	<0.001
%fPSA	14.67±7.27	19.06±7.52	<0.001
p2PSA	19.55±14.93	18.68±12.46	0.779
%p2PSA	2.39±1.35	1.61±0.62	<0.001
PHI	54.77±31.21	39.15±15.59	<0.001
Protein	77.10±4.74	78.10±4.85	0.252
CRP	1.90 (1-3.8)	1.75 (0.9-3.3)	0.532
Testosterone	19.18±6.93	18.63±6.19	0.795

*Med (25^th^ -75^th^ percentile)

The distribution of the PSA value category according to the presence of PC is shown in Table-2. According to this analysis, there was no statistically significant difference in this variable among patients with and without presence of this malignancy (p=0.820).

**Table 2:** Distribution of patients with or without prostate cancer according to total prostate specific antigen.

Total PSA	Prostate cancer	

	No	Yes
2-2.9	4 (6.2%)	5 (7.7%)
3-3.9	6 (9.4%)	8 (12.3%)
4+	54 (84.4%)	52 (80.0%)

No significant difference between groups (p=0.808)

Table-3 represents area under the curve (AUC), cut off values and sensitivity and specificity for each chosen cut off value. Left side of the table represents AUC for all patients (PC and controls) while right side of the table represents AUC only for patients with PC. When analyzing diagnosis of PC, the highest area was observed in %p2PSA, following by fPSA and %fPSA, while the lowest observed in tPSA. We presented three cut-off values for %p2PSA and PHI because no adequate cut off was obtained on ROC graph. But, of those three variants, best ratio of sensitivity and specificity for %p2PSA would be at cut-off 1.67 and 41.67 for PHI. When analyzing diagnosis of GS ≥7 only PHI and %p2PSA are significant (PHI is almost significant, very close to conventional level of significance, 0.05). Same as for diagnosis of PC, three possible cut-off values for PHI and %p2PSA are present.

**Table 3:** Area under the curve AUC.

	Controls vs Prostate cancer (n=129)					Gleason <7 vs ≥ 7 (n=65)				
	
	Area	p value	Cut off	Sn	Sp	Area	p value	Cut off	Sn	Sp
tPSA	0.563 (0.464-0.662)	0.215	3.47	90.6	9.2	0.538 (0.389-0.687)	0.608	3.530	92.0	10.0
			5.55	62.5	49.2			5.275	64.0	45.0
			8.42	15.6	90.8			8.175	16.0	90
fPSA	0.707 (0.617-0.798)	<0.001	0.550	90.6	29.2	0.520 (0.375-0.664)	0.793	0.400	90.0	16.0
			1.035	60.9	81.5			0.665	67.5	40.0
			1.520	21.9	90.8			1.525	10.0	92.0
%fPSA	0.693 (0.602-0.785)	<0.001	11.410	90.6	40	0.529 (0.386-0.671)	0.701	6.825	90.0	8.0
			12.905	81.3	49.2			11.24	65.0	60.0
			22.565	25.0	90.8			21.95	15.0	92.0
p2PSA	0.514 (0.414-0.615)	0.779	8.205	90.6	13.4	0.581 (0.436-0.726)	0.275	7.770	92.0	15.0
			13.020	60.9	61.5			15.24	64.0	40.0
			32.160	9.4	90.8			28.41	16.0	90
PHI	0.680 (0.588-0.772)	<0.001	27.480	90.6	26.6	0.645 (0.505-0.784)	0.054	31.33	91.7	22.5
			41.670	64.1	62.5			49.13	66.7	60.0
			61.015	28.1	90.6			76.38	25.0	90
%p2PSA	0.723 (0.632-0.810)	<0.001	1.245	90.8	34.4	0.673 (0.534-0.812)	0.020	1.356	92.0	20.0
			1.673	75.4	64.1			2.495	56.0	17.5
			2.368	43.1	90.6			3.076	32.0	90

Univariate and multivariate logistic regression were used to assess predictive value of PSA isophorms (Table-4 and Table-5). In whole sample model, univariate analysis revealed that fPSA, %fPSA, PHI and %p2PSA are significant predictors of PC. Also, %p2PSA has highest R^2^ which suggests that it is the best marker for PC. In multivariate model, p2PSA, PHI and %p2PSA are significant predictors of PC. In PC group, %p2PSA is significant (PHI and p2PSA are almost significant, very close to conventional level of significance, 0.05).

**Table 4:** Univariate model for prostate cancer prediction and ≥ 7 Gleason score.

	Controls vs Prostate cancer (n=129)				Gleason <7 vs ≥ 7 (n=65)			
	
	P value	OR (95% IP)	R^2^	H-L^a^	P value	OR (95% IP)	R^2^	H-L^a^
tPSA	0.219	0.894 (0.749-1.069)	0.016	0.925	0.663	1.058 (0.820-1.367)	0.004	0.946
fPSA	0.001	0.247 (0.110-0.553)	0.148	0.025	0.547	0.700 (0.220-2.232)	0.008	0.040
%fPSA	0.002	0.920 (0.872-0.970)	0.111	0.604	0.419	0.970 (0.902-1.044)	0.014	0.506
p2PSA	0.737	1.004 (0.981-1.028)	0.001	0.548	0.116	1.028 (0.993-1.063)	0.061	0.185
PHI	0.001	1.037 (1.015-1.060)	0.146	0.993	0.052	1.021 (1.000-1.042)	0.102	0.983
%p2PSA	<0.001	3.016 (1.715-5.305)	0.207	0.574	0.024	1.880 (1.086-3.256)	0.150	0.869

^**a**^ Hosmer and Lemeshow test p value

**Table 5:** Multivariate model for prostate cancer prediction and ≥ 7 Gleason score.

	Controls and Prostate cancer (n=129)				Gleason <7 and ≥ 7 (n=65)			
	
	P value	OR (95% IP)	R^2^	H-L^a^	P value	OR (95% IP)	R^2^	H-L^a^
tPSA	0.394	1.175 (0.811-1.702)	0.160	0.247	0.589	1.137 (0.714-1.809)	0.020	0.932
fPSA	0.074	0.152 (0.019-1.198)			0.631	0.505 (0.031-8.201)		
%fPSA	0.851	1.011 (0.901-1.135)			0.928	1.007 (0.857-1.185)		

tPSA	0.385	1.178 (0.814-1.705)	0.266	0.118	0.583	1.142 (0.711-1.835)	0.148	0.949
fPSA	0.006	0.042 (0.004-0.407)			0.204	0.131 (0.006-3.013)		
%fPSA	0.767	1.018 (0.907-1.142)			0.824	1.019 (0.864-1.202)		
p2PSA	0.007	1.086 (1.023-1.152)			0.058	1.059 (0.998-1.125)		

tPSA	0.939	1.015 (0.689-1.495)	0.268	0.358	0.879	1.039 (0.636-1.696)	0.116	0.554
fPSA	0.131	0.208 (0.027-1.598)			0.600	0.461 (0.025-8.358)		
%fPSA	0.744	1.019 (0.908-1.145)			0.828	1.019 (0.863-1.203)		
PHI	0.004	1.037 (1.012-1.064)			0.065	1.021 (0.999-1.044)		

tPSA	0.396	1.179 (0.806-1.725)	0.279	0.044	0.532	1.165 (0.721-1.882)	0.163	0.562
fPSA	0.135	0.211 (0.027-1.623)			0.614	0.477 (0.027-8.486)		
%fPSA	0.760	1.018 (0.906-1.145)			0.808	1.021 (0.864-1.205)		
%p2PSA	0.003	2.451 (1.361-4.414)			0.027	1.848 (1.071-3.189)		

^**a**^ Hosmer and Lemeshow test p value

## DISCUSSION

Early detection of PC remains the most important issue for general practitioners, patients, researchers, and the experts in the field of urology. During the past decades, efforts are being made to identify tools or biomarkers that can maximize early diagnosis of aggressive disease, but curable, and minimize the undesirable effects of treatment of indolent disease.

In our study, we examined the relationship between PC (presence and aggressiveness according to the value of Gleason score) and the level of the PSA, and its derivates, especially %p2PSA and PHI. To the best of our knowledge, this kind of investigation is the very first one conducted in Balkan population. According to results of our study, investigated biomarkers could distinguish benign from malign changes in prostate and between high and low malignant potential tumor changes in patients with confirmed PC. The findings in our study indicated that p2PSA, %p2PSA and PHI were independent predictors of this malignancy. Also, they showed promising predictive value for detection of high malignancy potential, especially %p2PSA which achieved the best results and statistical significance. Other two are near statistical significance and it is very likely that larger sample size could provide significance. Also, in multivariate models, it has been shown that inclusion of p2PSA, %p2PSA and PHI increased prediction of PC presence and level of its aggressiveness (assessed by Gleason score), although p2PSA and PHI did not reach conventional level of statistical significance in Gleason score groups (<7 and 7+), but p values are very close to 0.05.

During the past years numerous studies have been performed in other to explore the predictive value of different biomarkers in PC detection (15-22). Le et al. showed evidence in distinguishing PC from benign disease using the %p2PSA in 2.034 men with PSA between 2.5 and 10ng/mL, normal DRE (20). Moreover, in their investigation, Stephan et al. showed that the PHI, the absolute value of 60, had greater power to predict clinically significant prostate cancer (Gleason ≥7) compared to p2PSA, %p2PSA, total PSA and %fPSA (21). Loeb et al. also presented evidence that supports the PHI to distinguish men with clinically significant prostate cancer (Gleason ≥7) compared to the tPSA (22). Therefore, the results from these studies consistently showed that p2PSA and its derivates represented improved and more reliable prognostic tools for PC detection, especially for those cases with Gleason score of 7 and more. It has been widely hypothesized that %p2PSA and PHI could be the best predictors of PC presence, with the significantly better accuracy than commonly used makers such as tPSA and %fPSA (11, 18, 20). The results from Serbian PC population confirmed and extended these findings and also provided further evidence that %p2PSA and PHI could be considered as power tools for improvement the accuracy in the early detection of clinically significant PC.

In our investigation, we used ROC and AUC analyses, as a part of comprehensive statistical approaches in assessing significant cut-off values of different potential PC biomarkers. Extensive employment of the existing literature led to the conclusion that %p2PSA and PHI have the highest AUC, leading to the hypothesis that these indicators represented the most promising predictors of prostate malignancy (10, 11, 23-26). According to these findings, PHI has the highest AUC, but very similar to %p2PSA. The results from our study have revealed that in our cohort of males %p2PSA represented leading PC biomarker, with the highest AUC. This potential predictive ability has been noted in both, discriminating benign from malignant prostate tumor, and more aggressive (Gleason score ≥7) from less aggressive forms of PC (Gleason score <7). Similar to other authors, PHI revealed high discriminating power, but less than %p2PSA, especially in discriminating high aggressive forms of PC from less aggressive forms. Results of our study also indicated high discrepancy between sensitivity and specificity of these markers. Similar to other researchers, 90% sensitivity is followed by low specificity and vice versa. Nevertheless, %p2PSA and PHI demonstrated the best ratio between sensitivity and specificity. Since ideal combination of sensitivity and specificity in our study is not available, we presented cut off values for combination of sensitivity and specificity of 90%, which is also suggested in others similar investigations (19, 26, 27).

With the aim to assess the independent predictors of prostate malignancy, we also performed the logistic regression analyses. The results from univariate logistic regression showed that %p2PSA and PHI had the highest predictive value for PC detection, as well as for distinguished clinical form with Gleason score ≥7 from the non-clinically significant one. The results from this type of analysis were in accordance with those obtained in ROC analysis. Namely, %p2PSA appeared to be a better indicator of malignancy than PHI, especially when aggressive form is the dependent variable. In this regression analysis PHI is also near conventional level of significance (p value is 0.054 in AUC analysis and 0.052 in logistic regression) and it is possible that higher sample size could reach statistical significance at conventional level. However, in multivariate model, %p2PSA remained significant predictor of PC and its aggressive forms, but PHI remained significant only in PC prediction. It is very important to note that in prediction of aggressive form, p value is higher in multivariate than in univariate model. Therefore, the addition of %p2PSA and PHI in multivariate model improved model itself, and made decision process more accurate, if based on this probability (21). Our results are in accordance with the findings from the other studies. Namely, a few prospective multicenter studies demonstrated that the %p2PSA and PHI have an improved prediction of clinically significant PC, both in men with a PSA between 4-10ng /mL and between 2-10ng/L (10-13, 15, 18). These biomarkers may therefore also have a role in monitoring men under active surveillance.

Some limitations of the present study need to be kept in mind in the interpretation of the results. First, this investigation was performed at a single institution, thus, results may not be generalizable to other health-care settings. However, the consecutive sampling design, in defined period of time, ensures the representativeness of the sample and the generalizability of the results. Secondly, cross-sectional design captures association but does not allow for causality or temporal sequence to be assessed. Moreover, keeping in mind the fact that group of patients without PC was also selected from the cohort of patients who visited the urologist and underwent prostate biopsy during the period of investigation, some kind of selection bias could also be introduced. Nevertheless, such kind of sampling to assess predictive value of different tumor markers in detection of patients with aggressive PC (assumed as Gleason score ≥7) in a subcohort of men whose total PSA ranged from 2 to 10ng/mL, supported the investigated hypothesis. Finally, agressivness of the PC has been estimated only by assessing the Gleason score which is only one of the criteria for the aggresive tumor potential. It is clear that more comprehensive estimation of tumor-related agressiveness should include other parametes such as: number of sections with cancer, percentage of cancer in a single section etc (27, 28).

## CONCLUSIONS

Derivates of p2PSA, PHI and especially %p2PSA, represented potentially very important clinical tools for predicting presence of PC in a cohort of Serbian males. Even more, these new markers could discriminate patients with Gleason score <7 from those Gleason score ≥7, within total PSA ranging from 2 to 10ng/mL. Those findings are central to avoid over diagnosis and subsequent over treatment.

## ARTICLE INFO

Int Braz J Urol. 2017; 43: 48-56
